# Challenges in recruiting African-American women for a breast cancer genetics study

**DOI:** 10.1186/s13053-018-0091-3

**Published:** 2018-04-24

**Authors:** Amanda J. Compadre, Melinda E. Simonson, Katy Gray, Gail Runnells, Susan Kadlubar, Kristin K. Zorn

**Affiliations:** 1 Division of Gynecologic Oncology, College of Medicine, University of Arkansas for Medical Sciences, 4301 W Markham St. Slot 793, Little Rock, AR 72205 USA; 2 Division of Medical Genetics, College of Medicine, University of Arkansas for Medical Sciences, 4301 W Markham St., Little Rock, AR 72205 USA

**Keywords:** Hereditary breast cancer, African American, Subject recruitment, Biorepository

## Abstract

**Background:**

African-American women, especially in the southern United States, are underrepresented in cancer genetics research. A study was designed to address this issue by investigating the germline mutation rate in African-American women in Arkansas with a personal and/or family history of breast cancer. Women were tested for these mutations using a large panel of breast cancer susceptibility genes. In this analysis, we discuss the challenges encountered in recruiting African-American women from an existing biorepository to participate in this study.

**Methods:**

We attempted to contact 965 African-American women with a personal and/or family history of breast cancer who participated in Spit for the Cure (SFTC) between 2007 and 2013 and provided consent to be recontacted. The SFTC participants were invited by telephone and email to participate in the genetic study. Enrollment required completion of a phone interview to obtain a family and medical history and return of a signed consent form.

**Results:**

Among eligible SFTC participants, 39.6% (382/965) were able to be contacted with the phone numbers and email addresses they provided. Of these, 174 (45.5%) completed a phone interview and returned a signed consent form. Others were not able to be contacted (*n* = 583), declined to participate (*n* = 57), did not keep phone interview appointments (*n* = 82), completed the phone interview but never returned a signed consent (*n* = 54), were deceased (*n* = 13), or were too confused to consent to participate (*n* = 2).

**Conclusions:**

Recruiting African-American women into our breast cancer genetics study proved challenging primarily due to difficulty establishing contact with potential participants. Given their prior participation in breast cancer research, we anticipated that this would be a highly motivated population. Indeed, when we were able to contact SFTC participants, only 14.9% declined to participate in our study. Innovative communication, retention, and recruitment strategies are needed in future studies to address the recruitment challenges we faced.

## Background

Breast cancer is the most commonly diagnosed cancer in African-American women with an estimated 30,700 new diagnoses in 2016 [1]. The overall incidence of breast cancer in African-American women is lower than the incidence in white women; however, African Americans have a mortality rate 39% higher than whites [1, 2]. African-American women are also more likely than other populations to be diagnosed at a younger age and have the more aggressive triple-negative type of breast cancer [3]. Poorer outcomes in African-American women appear to be related to screening rates, access to care, tumor biology, environment, and cancer genetics [4–6].

In the era of precision medicine, cancer genetics is being emphasized to better identify factors that underlie breast cancer disparities [7]. However, African-American women, especially in the southern United States, are underrepresented in cancer genetics research [6, 8, 9]. A study at the University of Arkansas for Medical Sciences was designed to address this issue by investigating the germline mutation rate in African-American women in Arkansas with a personal and/or family history of breast cancer using a multiplex panel from the University of Washington that includes 48 genes, including *BRCA1, BRCA2* and other known cancer susceptibility genes [10, 11].

Women were recruited for the genetic study using the established Spit for the Cure (SFTC) cohort. SFTC created a biorepository at the University of Arkansas for Medical Sciences of over 26,000 women’s saliva samples that were donated for breast cancer research. Women were recruited between 2007 and 2013 at community events across Arkansas, a state that is 43.8% rural according to census data [12, 13], and represent the demographic make-up of the state, with an oversampling of women with a history of breast cancer. The SFTC cohort represents a more diverse population than previously studied; for example, in 2011 the cohort had 15.8% African-American participants which corresponds closely to the 15% African-American population of Arkansas [12]. SFTC has 5495 African-American women enrolled.

SFTC participants completed a questionnaire including demographics and medical information, such as family history of breast cancer in first-degree relatives, personal history of breast cancer, treatment if the participant had breast cancer, reproductive history, BMI, alcohol consumption, and physical activity [12]. Approximately 24,700 SFTC participants indicated their willingness to be recontacted about future studies, including 5155 African Americans (20.9%). We felt this biorepository represented a unique opportunity to study the germline mutation rate in a relatively understudied population from the southern US.

## Methods

After receiving IRB approval, eligible SFTC participants were identified. Eligibility criteria included self-identification as an African-American woman, a personal and/or family history of breast cancer, and agreement to be recontacted for future research. For purposes of this study, family history of breast cancer was defined as having a first-degree relative with breast cancer. Participants with both a personal and family history of breast cancer were included in the personal history group. If participants were found to be from the same family, they were not excluded from enrolling. A total of 965 African-American SFTC participants, including 296 with a personal history and 669 with a family history of breast cancer, were considered eligible based on these criteria.

At least two and up to four attempts were made to contact each SFTC participant using the telephone numbers and/or email addresses they provided to SFTC. If the participant had an automated answering service, a voicemail with our contact information was left. If other members of the household answered, they were asked to give a message with our contact information to the participant. If SFTC participants were interested in our study and available when called, the phone interview was conducted immediately. If SFTC participants responded via email or expressed interest but could not complete the interview immediately, an appointment was made for the interview to be completed at the participant’s convenience. When scheduling appointments, the study team was available from 7 am to 8 pm on weekdays and weekends.

After confirming eligibility, the purpose of the research study was explained. This included counseling about the benefits and risks of having genetic testing, including the possible outcomes of genetic testing. Genetic counseling was provided and informed consent was obtained by a certified genetic counselor or a physician. Once a woman verbally consented to participate, a detailed family and medical history was obtained. Although we did not record interview duration, we estimate the average interview lasted 30 min but varied depending on the family size and complexity of their medical history. After the interview, a consent form was mailed with instructions to sign and return it within 2 weeks using a pre-addressed, stamped envelope. Participants were given a tentative timeline of when to expect results. They were informed that further genetic counseling would be available regardless of the results. If an abnormality was detected, confirmatory testing, counseling, and cascade testing would also be provided to participants and their potentially affected family members. Variants of unknown significance were not returned to participants; if these variants were to be reclassified to a pathogenic mutation in the future, every effort would be made to reach those participants Women who completed the phone interview but did not return a signed consent form within 1 month were called with a reminder to return the signed form. An additional consent form was also mailed.

## Results

When women initially enrolled in SFTC, they provided their education level, location (rural or urban), and age. In the 965 women that we attempted to contact in 2015, the average age of the women at attempted recontact was 55.8 years with a range of 25–91 years. The majority of the women in this subset of SFTC were located in urban areas (79.4%) versus in rural areas (20.6%) [Table 1].

**Table 1:** Demographics

	Enrolled (*n* = 174)		Not Enrolled (*n* = 791)		Total (*n* = 965)
	PH^a^ (*n* = 65)	FH^b^ (*n* = 109)	PH (*n* = 231)	FH (*n* = 560)	
Education					
Less than high school graduate	1	3	19	42	65
High school graduate or GED	11	12	52	128	203
Some college or technical school	23	30	92	170	315
College or post-college graduate	30	64	68	220	382
Location^c^					
Rural	13	14	43	128	198
Urban	52	95	188	431	766
Unknown	0	0	0	1	1
Age at attempted contact 2015 (years)					
Mean	61.5	53.2	60	53.9	55.8
Range	32–88	25–79	33–91	25–86	25–91

^a^Personal history of breast cancer

^b^Family history is defined as having at least one first degree relative with breast cancer

^c^Rural or urban as defined by the US Census 2010

Of the 965 SFTC participants, 364 provided a phone number(s) when they enrolled in SFTC, three provided an email address, and 598 provided both a phone number and email address. Of the 174 SFTC participants who enrolled in our study, 37 provided only their phone number, whereas 137 provided both a phone number and an email address. Overall, significantly fewer of those who provided only a phone number (10.2%) were enrolled into our study compared to those who provided both a phone number and an email address (22.9%; *p* < 0.01).

**Fig. 1:**
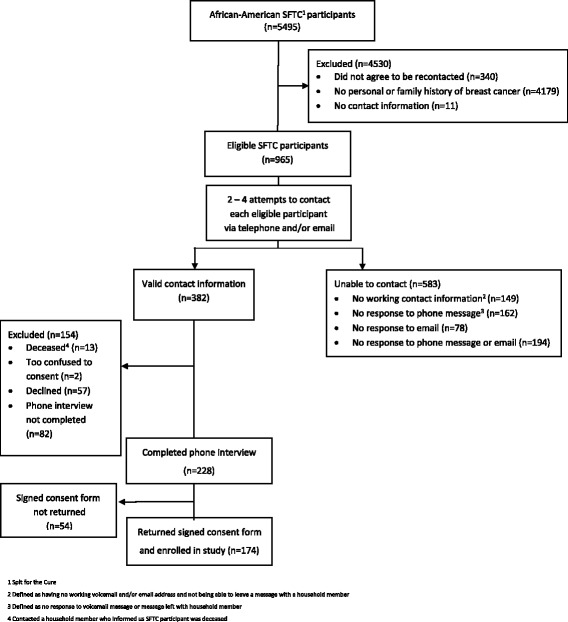
Recruitment

Among eligible SFTC participants, 39.6% (382/965) were contacted or accounted for with the phone numbers and email addresses they provided. Of these, 174 (45.5%) completed a phone interview and returned a signed consent form and thus were enrolled in the study. Reasons why other eligible subjects were not enrolled in the study included inability to contact them (*n* = 583), declining to participate (*n* = 57), not keeping scheduled appointments for phone interview (*n* = 82), completing the phone interview but never returning a signed consent form (*n* = 54), being deceased (*n* = 13) or being too confused to consent to participate (*n* = 2) [Fig. 1].

Initial enrollment in SFTC occurred between 2007 and 2013, while our recruiting efforts took place in 2015. To determine if the time between SFTC enrollment and our recruitment impacted participation, we compared the year women who enrolled in our study joined SFTC to the total number of women from each year eligible to join. We did not find a trend in enrollment rates per year. The percentage of women enrolling in our study each year ranged from 9.4% in 2010 to 25% in 2008 (data not shown).

Of the 296 African-American SFTC participants with a personal history of breast cancer, we were able to contact or account for 142 of them (48.0%). Of these, 85 completed a phone interview and 65 returned a signed consent form (Table 2). Among those who were contacted but did not participate, 22 agreed to participate but did not keep their phone interview appointment. For eleven of them, a household member informed us that the participant was deceased. Two were too confused to consent to participate and 22 declined. Overall, 154 participants with a personal history of breast cancer could not be accounted for or contacted (52.0%).

**Table 2:** Recruitment Results

		Total potential participants		Personal history of breast cancer		Family history of breast cancer^a^	
		Total	*(%)*	Total	*(%)*	Total	*(%)*
Valid contact information (*n* = 382)	Completed phone interview, returned consent form	174	*18.0*	65	*22.0*	109	*16.3*
	Completed phone interview, consent form not returned	54	*5.6*	20	*6.8*	34	*5.1*
	Did not complete phone interview	82	*8.5*	22	*7.4*	60	*9.0*
	Deceased^b^	13	*1.3*	11	*3.7*	2	*0.3*
	Too confused to consent	2	*0.2*	2	*0.7*	0	*0*
	Declined	57	*5.9*	22	*7.4*	35	*5.2*
Unable to contact (*n* = 583)	No working contact information^c^	149	*15.4*	43	*14.5*	106	*15.8*
	No response to phone message^d^	162	*16.8*	35	*11.8*	127	*19.0*
	No response to email	78	*8.1*	14	*4.7*	64	*9.6*
	No response to phone message and email	194	*20.1*	62	*20.9*	132	*19.7*
	Total	965	*100.0*	296	*100.0*	669	*100.0*

^a^Family history is defined as having at least one first-degree relative with breast cancer

^b^Contacted a household member who informed us the Spit for the Cure participant was deceased

^c^Defined as having no working voicemail and/or email address and not being able to leave a message with a household member

^d^Defined as no response to voicemail message or message left with household member

Of the 669 SFTC participants with a family history of breast cancer, 240 (35.9%) were contacted or accounted for. Of those, 143 completed a phone interview and 109 returned a signed consent form (Table 2). Among the 97 others who were contacted but did not participate, 60 agreed to participate but did not keep their phone interview appointment and 35 declined. Two were deceased according to a household member. Overall, 429 of the participants with a family history of breast cancer could not be accounted for or contacted (64.1%).

A significantly higher percentage of those with a personal history of breast cancer completed an interview compared to those with a family history of breast cancer (28.7% versus 21.3%, respectively; *p* < 0.01), but both groups had similar rates of returning signed consent forms after interview completion (76.4% and 76.2%, respectively). Therefore, a higher percentage of women with a personal history of breast cancer were ultimately enrolled into our study. A similar percentage of women with a personal or family history of breast cancer declined to be in the study (7% and 5%, respectively).

## Discussion

Barriers to minority recruitment in research are complex and multifactorial [9, 14, 15]. A study investigating the factors involved in decisions to participate in cancer genetics research found that many African-American participants had conflicting views about genetics research. They considered the potential benefit to themselves, their family, and their community as positive outcomes; however, they still had concerns about exploitation and distrust of the researchers’ motives [16]. Despite these concerns, 73% of African Americans said they were willing to participate in a cancer genetics study that involved donating a biological sample [17]. Another study looking at research perceptions and motivating factors surrounding African-American participation in breast cancer research found that most women had a positive view, recognized the benefits, and wanted to participate in breast cancer research [6].

Although distrust may have played a role in SFTC participants declining to participate in our genetics study, the main obstacle to recruitment was successful recontact. Although SFTC participants had previously given consent for their samples to be used for breast cancer research, we felt it was important to recontact them to obtain a separate informed consent for the new study because of the implications that genetic test results could have on clinical care for them and their families. Anonymous use of SFTC samples would not have allowed us to give participants their results, to provide confirmatory genetic testing, or to offer them and their families genetic counseling and cascade testing. In addition, recontacting them allowed us to obtain a more detailed family and medical history than the one provided to SFTC.

We anticipated that 60–80% of eligible African-American SFTC participants would enroll in our genetic study based on the their previously demonstrated willingness to participate in breast cancer research, suggesting they were more highly motivated than a typical population [18]. We also believed that their personal and/or family history of breast cancer would make them more likely to participate, since this has been linked to higher overall retention in previous cancer genetics studies [19]. Indeed, when we were able to contact SFTC participants, most were interested in participating in our study, with 81.2% initially agreeing to participate and either completing a phone interview or making an appointment to complete it at a later date. Only 14.9% actively declined to participate. These rates are similar to those seen for African Americans in other genetic studies [16, 20].

Of note, there was at least a two-year gap between the end of enrollment in SFTC and recontact for our study, but enrollment in our study remained low regardless of the length of time since SFTC participation. Prior to our study, the SFTC team conducted two efforts to recontact SFTC participants. Mailings were sent in 2008 and 2010 to a total of 15,000 SFTC participants. In these mailings, 2398 African-American women and 12,602 non-African Americans were included. Responses were received from 39% (935/2398) of the African-Americans compared to 58.7% (7391/12,602) of the non-African Americans, suggesting low rates of successful recontact of African-American SFTC participants as early as 2008 and 2010 (Unpublished data from personal communication with Gail Runnells on March 20, 2018). Furthermore, this demonstrates that African-American participants had lower rates of recontact compared to non-African Americans in the SFTC cohort. The 39% recontact rate of African-American women in 2010 mirrors our experience, where we were able to account for 39.6% (382/965) of SFTC participants.

When contact was made, we had the most success with enrollment when participants were able to complete the interview immediately. We lost 82 potential enrollees when future scheduled appointments were not kept. The length of the interview therefore negatively impacted enrollment, but we consider that unavoidable as the information gathered in the interview was critical to the goals of the study. Other studies have shown that shortening the data collection portion of enrollment and/or allowing some of this process to be available online positively impacts recruitment [21]. Additionally, offering a monetary incentive may have increased enrollment [21, 22]. Although we did not offer a monetary incentive for participating, we did offer the incentive of free genetic counseling and testing.

Once the interview was completed, 76.3% (174/228) of participants signed and returned a consent form. The failure of 54 potential participants to return the consent form could represent passively declining to participate. Over the phone, some participants might not have fully understood the purpose, risks, and benefits of the study and were hesitant to participate once they saw them written in the consent. However, forgetting to return the consent forms, not understanding the importance of returning a signed consent form, or mailing errors could also account for the lack of signed consent forms. These are all commonly encountered issues in studies involving renewing consent [23]. In the future, more immediate ways of obtaining informed consent, including email and/or text messages, should be explored. This “participant-centered” way of communicating with potential subjects could streamline the recruitment and consent process [24].

Strategies are needed to overcome the barrier of low recontact rates for future studies. When planning a biorepository where minority participation is emphasized, one strategy to overcome low recontact rates could be to oversample minorities. Although SFTC recruited a proportional number of African Americans compared to Arkansas demographics and oversampled women with a personal and/or family history of breast cancer, specifically oversampling African-American women could have aided our recruitment efforts. Other studies have also suggested oversampling as an effective way to increase the number of individuals who participate [20]. Another study design consideration is the role distrust of the medical community could have played, which we are not able to formally assess because we did not record the reasons why SFTC participants declined to participate in our study. However, for future studies it is important to incorporate proven strategies that build trust between the participants and researchers such as community involvement, culturally sensitive and competent research protocols, and communication with participants during and following the completion of the study [6, 9, 15].

Another strategy to increase success of recontact attempts is collecting more detailed contact information when enrolling participants. Multiple modes of contact, including a secondary contact person, have been shown to be effective tools for retention in cohort studies [21, 22]. In our study, women who provided both a phone number and an email address (22.9%) were statistically more likely to be enrolled compared to those who provided a phone number alone (10%).

Increased frequency of contact and quality of interactions between the researchers and participants is an additional strategy to consider for future studies. We made between two and four attempts to contact each woman. Each additional attempt netted successful contacts, but we did not keep a detailed record of the exact increase in yield. A genetic counseling study with 192 women found that most of the African-American women who were retained in the study were reached after about five call attempts and that calling in the evening netted a higher yield [19]. Although we did recruit participants until 8 pm, limitations on resources did not allow us to attempt to contact each of the 965 more than four times. Additionally, we used publically searchable databases to get accurate contact information for a small subset of women who were eventually enrolled into the study. We prioritized SFTC participants who were at the highest risk for genetic mutations based off of their self-reported personal history of breast cancer. Limitations on time and resources did not allow us to publically search all 583 unreachable participants, though. Searching for contact information in databases such Lexus Nexus and the White Pages would have likely increased our enrollment numbers; future studies should consider incorporating the time and resources necessary for these tools in their planning. Finally, more frequent attempts to contact participants on behalf of SFTC may have increased our participation. A study looking at retention rates for African-American women enrolled in a longitudinal genetic counseling research study found that after one and 6 month follow-up telephone calls, 65% and 60% of women, respectively, were retained in the study [19]. The 2008, 2010, and 2015 efforts to reach SFTC participants may have left too long of a gap between enrollment and recontact. Finally, research shows the importance innovative retention strategies, including the use of social media and newsletters with an emphasis on study benefits [21, 22]. Again, both of these strategies require an additional investment of staff and resources, especially with a large cohort size.

## Conclusions

We encountered challenges recruiting African-American women with a personal or family history of breast cancer from an existing biorepository to participate in a study evaluating germline genetic mutations, despite the fact that they had previously participated in breast cancer research. When we were able to contact SFTC participants, only 14.9% declined to participate in our study. However, recruiting women into our study was hampered by difficulty contacting the subset of SFTC participants eligible for our study. Invaluable resources like the SFTC biorepository can be maximized for future studies by employing innovative strategies for maintaining participant engagement and up-to-date contact information, as well as allocating adequate resources for intensive contact attempts in subsequent studies.

## Abbreviations

SFTC: Spit for the cure
